# Fostering hand disinfections in medical facilities: discussion of ethical conflicts and technical solutions

**DOI:** 10.1186/s12910-026-01621-1

**Published:** 2026-09-22

**Authors:** Frank Russow, Clemens H. Cap, Johann-Christian Põder

**Affiliations:** 1 Institute of Computer Science, University of Rostock, Rostock, Germany; 2 Faculty of Theology, University of Rostock, Rostock, Germany

**Keywords:** Healthcare-associated infections, Hand hygiene monitoring, Infection prevention, Hand disinfection, Ethical conflict

## Abstract

**Background:**

Consistent compliance with hand hygiene is a crucial measure for the prevention of hospital-acquired infections. In recent years, various systems have been developed to support both nurses and doctors with improving their hand hygiene. However, the data sets collected to provide feedback offer the potential for excessive surveillance and punitive measures in cases of non-compliance, which creates an ethical conflict between promoting patient safety and preserving an ethically acceptable work environment for healthcare workers.

**Method:**

By adopting an interdisciplinary socio-technical approach and correlating the ethical concerns with the technological specification of such systems, the paper analyses this ethical conflict in detail.

**Results:**

The paper demonstrates that optimized implementation strategies can minimize the ethical tensions. Additionally, the authors point out several aspects of the design process of such monitoring systems, including the active participation and involvement of staff, which positively contribute to the mitigation of this ethical conflict and also increase user acceptance of the systems. It also covers the deployment of a monitoring system in a hospital in Stralsund, Germany and discusses previous observations while simultaneously highlighting the problems with the design of the system according to the described ethical tensions.

**Conclusion:**

While the installation of a hand hygiene monitoring system can benefit the awareness for hospital hygiene, the development of such systems remains an ongoing and evolving process, particularly regarding the resolution of the ethical conflict. This paper argues that such systems should be used in a non-punitive and transparent manner, developed participatively with medical staff, and implemented in ways that ensure the collected data are used positively and responsibly.

## Introduction

According to a study by the Robert Koch-Institut (RKI), roughly 500,000 people in Germany (approximately 2.5% of all patients [1]) suffer from a hospital-acquired infection (HAI) during their stay in medical facilities every year [2]. An infection is classified as such if it is acquired during a hospital stay and the first symptoms manifest at least 48 hours after the time of hospitalization [3]. Beyond causing additional suffering for the patients, these infections also increase hospital expenditures due to the necessity of extended treatment. Furthermore, the RKI estimates that around 15,000 people die every year as a result of a HAI [2].

Correct hand hygiene is recognized as the primary measure for preventing the transmission of multi-resistant pathogens [4]. The World Health Organization (WHO) identified five indications of necessary hand hygiene moments, where a hand disinfection is needed. However, these necessary hand disinfection procedures are not always performed. This represents a major problem, as strict compliance with hand hygiene protocols is essential to prevent the spread of infections [5].

To address this issue, the DiSH-O-Klin^1^ research project^2^ presented in this paper is researching and developing a system that is intended to foster hand hygiene in medical facilities by implementing various incentive mechanisms. This project builds upon Helicoph^3^ – a system that monitors the daily disinfections carried out by the medical staff which has already achieved initial results [6] – and expands it with new features intended to further improve the hygiene situation.

A key objective of the DiSH-O-Klin research project is the implementation of technical reminders to inform medical staff of the need for hand disinfections during their shifts. Comprehensive tracking of movements in the hospital and the tracking of individual actions during the workflows of each person will be required for a correct implementation. If all collected data records are stored, an analysis on the aggregated data would theoretically be able to derive answers to the following exemplary questions:

- Which nurse has disinfected their hands the least over a certain period of time?
- Which doctor ignores the reminders for disinfections most often?
- Which medical staff member disinfects their hands the least after using the toilet?
- Which doctor is less likely to disinfect their hands before entering a patient room?
- Which nurse or doctor did not disinfect their hands on the 25th of April at 3:56 PM when preparing an injection for the patient Mr. John Doe in room 528?

This paper examines how the possibility of answering these questions brings into focus a broader ethical conflict between protecting patient health and maintaining a good work environment for nursing staff. In the following, this ethical conflict will be analyzed in further detail. After that, the functionality of the existing Helicoph system is described in detail and evaluated within this conflict. To justify the necessity of such a system, initial results are presented. Finally, potential extensions to the system are discussed, alongside an examination of how a technical realization can be achieved in compliance with the precautions proposed in this paper in regards to the ethical conflict.

## Ethical conflict – a first glimpse

The introduction of a system that continuously monitors hand hygiene promises a significant benefit: enhanced protection for patients against HAIs. At the same time, however, such a system may undermine trust between employers and employees. It may also create fear of punishment or even dismissal if the rules for hand hygiene are not strictly followed. This presents a genuine ethical conflict: a measure introduced in the name of patient safety may also be perceived as a form of surveillance and have negative consequences for a good and ethically acceptable work environment.

This conflict can be explored from different perspectives. In the following, three possible ways of understanding digital hand hygiene monitoring will be distinguished: first, as a means of identifying responsibility (“guilt”) in cases of infection; second, as a basis for disciplinary intervention; and third, as an assistive tool intended to support reflection, training and behavioural change without punitive consequences.

### Hard case – solving the question of guilt

While the incubation period and transmission routes cannot always be clearly determined [7], a strict monitoring of the hand disinfections carried out could give the ability to identify possible culprits in the event of a serious illness or death of a patient due to a HAI.

This refers to a group of medical staff who (possibly) caused the infection and thus possibly the death of the patient by failing to disinfect their hands and therefore not adhering to hygiene standards. Whether such a simple determination of causality is possible, however, appears doubtful – HAIs arise in a multifactorial context in which numerous potential sources of infection come into question [7].

### Measures in the form of disciplinary punishment

Another question is whether extensive tracking or data collection should be used for disciplinary measures instead of directly assigning the blame. Even if the use of punitive disciplinary measures and their (detailed) discussion appear to be rather rare in the literature on digital hand hygiene systems, an ethical justification of a punitive element – especially with regard to the high ethical good or importance of patient safety – cannot be completely ruled out.

For example, the study [8] describes a package of measures for hand hygiene compliance that, in addition to training and support measures, also included a punitive element in the form of a “violation letter” based on human observation – with positive results. Nevertheless, punitive measures and the punitive use of digital tracking systems for hand hygiene remain controversial and are often seen as counterproductive. They also contradict the direction of the WHO hand hygiene strategy [4], which recommends a positive, supportive safety and quality culture.

### Monitoring to assist – without penalties

A third form of justification for the use of hand hygiene tracking systems and digital data collection, which is often discussed positively in the literature [9–11], lies in their function to support the nursing staff and remind them when a disinfection of their hands is necessary. Digital systems can raise awareness of hand hygiene, promote the empowerment of caregivers and provide individual feedback on hand hygiene behavior [12, 13].

At an institutional level, the collected data is used to plan and implement educational measures, identify problem areas and develop targeted support strategies. This form of intervention is not aimed at punitive disciplinary measures, but at support, reflection and positive behavioural change (e.g. through a positive change in professional self-image or organizational culture).

Even if digital hand hygiene monitoring is used in an assistive rather than punitive way, the ethical concerns do not disappear. The evaluation of such systems depends not only on their intended function, but also on their potential unintended effects on healthcare workers and on the institutional and structural context in which they are implemented. This includes the perspective of employee representation, the working conditions of nursing staff, and the normative aspects of a good work environment.

The multi-layered concept of a “good work environment” is understood primarily from an ethical perspective, with particular attention to its associated normative dimensions. It builds on broader occupational and healthcare literature in which a good work environment is understood as the result of several interrelated factors, including “demands, control, reward, fairness, leadership, communication, influence, social support, work-life balance, and appropriate skills” [14].

At the same time, the concept is informed by related healthcare literature on ethical practice environments, ethical climate, and healthy work environments [15–17]. In the context of digital hand hygiene monitoring, it is applied specifically to the normative workplace implications of digital tracking. For instance, minimizing stress and anxiety constitutes an organizational ethical requirement [18] that contributes significantly to a good work environment. Other ethical threats to a good work environment include the erosion of trust and the perceived loss of professional autonomy or privacy. A more precise exploration of these aspects allows for a more differentiated view (and weighting) of the ethical conflict. This includes recognizing that a poor work environment – often associated with punitive measures – can itself have problematic effects on patient safety [19].

## A closer look at the ethical conflict

The previous section provided a first overview of the ethical conflict surrounding digital hand hygiene monitoring. Building on this initial mapping, the following section takes a closer look at central ethical concerns discussed in the literature, especially privacy, surveillance, professional autonomy, and trust.

The number of studies investigating the effectiveness of digital monitoring systems to improve hand hygiene in medical environments is constantly increasing. For example, the OPTICOMS study (2024-2026)^4^ at the University of Göttingen is currently actively investigating the potential of such electronic aids. Overall, international studies and discussions suggest that electronic monitoring systems can improve hygiene and reduce the risk of infection, although the need for further research is emphasized [13, 20]. However, a growing body of literature also addresses the ethical aspects and user acceptance of electronic hand hygiene monitoring systems [9, 11, 21]. These studies highlight concerns such as privacy, autonomy, trust, and acceptance, which are taken up in the following analysis.

The ethical analysis follows a principle-oriented approach within a socio-technical perspective [22]. The principles of biomedical ethics developed by Beauchamp and Childress provide a general normative orientation, but are not treated as an exhaustive or fixed checklist [23, 24]. Rather, the relevant normative considerations were specified in dialogue with the empirical and ethical literature on digital hand hygiene monitoring. This resulted in particular attention to patient safety, privacy, professional autonomy, trust, data protection, and the structural conditions of care work.

The literature was identified in a purposive, problem-oriented manner rather than through a systematic review. Existing reviews of digital hand hygiene monitoring [11–13], together with broader empirical-ethical studies on ethics, acceptance, and user experience [9, 21], were selected as starting points because they provided an overview of recurring ethical concerns, technical characteristics, and practical experiences with such systems. These initial sources were then used to identify the main problem areas relevant to the present analysis. Additional literature was then selected to address specific issues, including privacy and surveillance, autonomy and nudging, trust and technological accuracy, data protection, employee participation, and structural working conditions.

In a further analytical step, these normative considerations were related to the technical characteristics of the hand hygiene monitoring technology under discussion. The analysis examines the relevant system characteristics, their intended behavioural or organizational functions, possible effects on healthcare workers, the ethical concerns they raise, and corresponding technical or organizational safeguards. In this way, it moves between technical design and normative evaluation, showing how particular design choices shape the ethical profile of the hand hygiene monitoring system in its socio-technical context.

### Privacy and surveillance

As mentioned above, the development and implementation of digital monitoring systems to improve hand hygiene are motivated by concerns for patient safety, which are closely related to the central medical ethical principles of non-maleficence [11] and beneficence. Avoiding patient harm and increasing patient safety are strong arguments in favor of using such systems. In contrast, aspects or problem complexes such as surveillance, privacy and erosion of trust are often cited in the research literature as ethically relevant concerns about such systems [12]. In [10], digital hand hygiene systems were viewed with skepticism by some participants as they could lead to constant monitoring and data collection by management and thus represented an “intrusion into their privacy”, even though the technology was very well accepted by most participants in the study.

The collection of movement data to detect disinfection processes can indeed reveal sensitive behavioral patterns. It is therefore necessary to reduce the data collected to a minimum in order to minimize the invasion of privacy. Another important factor to this is the purpose limitation of the gathered data [12, 25], where access and processing should be strictly limited.

For example, the timestamps of the recorded hand disinfections could possibly provide information about compliance with working hours or the pace of work [26, 27]. In order to not create performance- and surveillance-related pressure on the staff members, the use of the data for purposes other than optimizing hand disinfection procedures should be ruled out.

### Professional autonomy and erosion of trust

Only a few studies have looked at the effects of such monitoring systems on the professional autonomy of care professionals so far [9]. One possible perspective is that the use of these technologies supports medical staff, but at the same time restricts their freedom of choice. As workflows can vary depending on the patient, the system must be highly flexible.

However, the relationship between tracking systems and autonomy can also be understood differently. Drawing on an overview of the ethics of nudging by [11, 28] argues that hand hygiene tracking systems can function as persuasive technologies and, more specifically, as “nudging instruments”. Strictly speaking, however, only certain features qualify as nudges, such as reminders or private feedback to the individual staff member. They count as nudges when they predictably influence behaviour by shaping the choice architecture while leaving all relevant options open and avoiding coercion or significant incentives [28, 29]. By contrast, the tracking and collection of data are forms of monitoring rather than nudging; publicly visible feedback may create social pressure, and the use of data to compare, evaluate, or sanction staff constitutes performance management or direct behavioural control.

From this perspective, a well-designed nudging strategy can support rather than merely restrict autonomy by helping caregivers to act in accordance with professional ethical goals they already endorse, such as patient well-being and patient safety [9, 11]. However, a beneficial aim alone neither makes an intervention a nudge nor suffices to justify it ethically. Future research should evaluate when “digital choice architecture” truly supports professional autonomy, while addressing its ethical risks and drawbacks [28, 30, 31].

### Data protection

Digital hand hygiene monitoring raises data protection concerns because it may involve the collection of identifiable information about healthcare workers, such as disinfection records, timestamps, or movement data. Depending on their content and linkage, such data may fall into different legal categories, including ordinary employee personal data, patient-related data, or, where health information is revealed, special categories of personal data under Article 9 GDPR. Their processing must therefore comply with the applicable requirements of the GDPR and national employment-data law, including requirements of purpose limitation, data minimisation and necessity (Art. 5 GDPR), as well as appropriate safeguards in the context of workplace monitoring (Art. 88 GDPR).

From an ethical perspective, however, legal permissibility alone is not sufficient. The central question is whether the collection and use of data are proportionate to the aim of improving patient safety, compatible with privacy, professional autonomy, and trust, and supported by appropriate governance and employee participation. This requires limiting data collection to what is genuinely necessary, restricting access and secondary uses, and avoiding forms of individualized monitoring that may create undue pressure or enable punitive use. Where possible, anonymized or sufficiently aggregated data should therefore be preferred, particularly when the purpose is quality analysis, training, or the development of supportive measures.

### Structural conditions

The implementation of such solutions for monitoring the hand hygiene, even when intended as assistive tools, faces considerable criticism from the works councils in the hospitals [32]. In Germany, according to the Betriebsverfassungsgesetz (Works Constitution Art), works councils have a right to co-determination regarding the introduction of technical solutions designed for monitoring the staff during their work (Article 87(1)(6))^5^. Their primary objective is to prevent intrusive surveillance and to maintain a pleasant and positive work environment.

In 2017, the ver.di (United Services Trade Union in Germany) launched an initiative calling for nursing staff in all medical facilities to strictly follow all hand hygiene requirements and recommendations [33]. Many hospitals were forced to terminate this experiment after only a few hours, as they were unable to keep up with the number of patients due to labour shortage [33].

Indeed, [33] argues that the hygiene issue does not stem from incorrect application or ineffective training, but rather from flawed assumptions regarding workforce capacity. This highlights a broader structural problem: non-compliance cannot always be adequately understood as individual failure, as it often results from working conditions that make full compliance difficult in everyday practice.

Taken together, these considerations show that the ethical evaluation of digital hand hygiene monitoring cannot be reduced to technical effectiveness alone. It must also address privacy, professional autonomy, trust, data protection, and the structural conditions of care work as integral ethical dimensions of a healthy work environment.

## Ethics and acceptance: digital monitoring systems for hand hygiene – not “If”, but “How”

Considering the growing number of studies indicating the effectiveness of digital hand hygiene monitoring systems, their use cannot be ruled out in principle on ethical grounds. The central question therefore seems to be less whether such systems should be used at all than under which conditions they can be designed, implemented, and integrated into clinical practice in an ethically justifiable way. Some studies show that medical staff is generally accepting a technology that helps to promote hand disinfection [11, 21]. However, the specific technology used in [11], for example, was only moderately accepted, which was mainly caused by inaccuracies in the measurements and the lack of organisational support [11].

To ensure that the monitoring system is accepted by medical staff, it is essential to identify which processes and methods in the development, implementation, subsequent analysis and evaluation of the collected data sets lead to a high level of user approval. The following considerations therefore appear to be particularly important for the development of a hygiene monitoring system:

### Transparency and co-determination

The nursing staff should be fully informed about the system’s functionality. This includes the type and the amount of data that is recorded and stored, as well as who may access this data and under what circumstances [12]. However, in the workplace context, transparency alone is not sufficient. Because employees may have limited ability to refuse the use of such systems, their acceptance and legitimacy should be secured through transparent procedures and collectively negotiated conditions involving works councils or other relevant employee representation bodies [34]. Implementing such systems requires negotiated agreements that define purpose, access, and safeguards against excessive or punitive surveillance.

### Participatory development and organizational support

As [30] points out, it is important to involve and encourage caregivers to participate actively in the design of such a digital innovation, as this fosters trust in both the digital system and the implementation process. Furthermore, the learning phase associated with interaction with the new system must be intensively supported.

It is crucial that nursing staff perceive the development and use of such technology as a shared responsibility, integrating it into their professional self-perception (in the service of patient safety), organizational culture and practical work processes. Only such a multidimensional “fit” enables the successful acceptance of such a technology [35].

### The question of accuracy

The accuracy of a hand hygiene monitoring system is critical to its acceptance among nursing staff [21]. When performed disinfections are incorrectly counted or when there are missed indications for a disinfection that should not have triggered one in the first place, the trust in the system is lowered, leading to a decrease in motivation to comply with the prompts from the system [21]. One potential solution is to employ a more precise distance measurement technology, such as Time Difference of Arrival (TDoA) via acoustic signals, instead of relying on the signal strength of wirelessly transmitted data packets [36].

### Positive use of data

The nursing staff often fear that monitoring systems of this kind may be used punitively [25]. To prevent a negative work environment, medical institutions should define and communicate, in collaboration with works councils or other relevant employee representation bodies, how data is handled (e.g. for awareness, reminders, empowerment, teamwork, quality analysis, training, or supervision).

Where data is used at group level, it should be aggregated in a way that prevents re-identification or quasi-individual tracking, especially in small teams or specific shifts. If the data indicates low compliance in a particular area or period, the administration should not interpret this finding in isolation, but in cooperation with the affected staff to identify causes and solutions together. Such a joint investigation can mitigate the feeling of surveillance and support the development of constructive measures.

### Consistent data protection

Although data records on hand disinfection procedures are not as sensitive as medical records from the patients, they still contain personal data regarding medical staff, making robust IT security essential [12]. Consequently, the data must be encrypted and stored securely. It is also recommended to delete the data to prevent misuse when it is no longer needed for analysis and evaluation.

## Helicoph – first practical experiences

The preceding ethical analysis has shown that digital hand hygiene monitoring cannot be evaluated solely in terms of technical effectiveness. Its ethical acceptability also depends on whether the system protects privacy, supports professional autonomy, avoids excessive or punitive surveillance, involves employee representation, and addresses the structural conditions of care work. Against this background, this chapter turns to a concrete example of such a system and examines how these considerations are reflected in its practical development and implementation.

The Helios Klinikum in Stralsund, Germany, developed a hand hygiene monitoring system called “Helicoph”, which aims to improve the hygiene situation in medical facilities, thereby creating a safer environment for both the nursing staff and the patients [6, 36]. In the following, the incentive mechanisms designed to encourage staff to disinfect their hands more frequently will be described and discussed. Furthermore, initial findings [6] by the clinic after a ten-month long testing phase will be presented and critically discussed. Concluding this section, current limitations are identified, followed by approaches to system extensions to address these challenges and issues regarding the ethical conflict.

### Smart name badge as the most important incentive

**Fig. 1:**
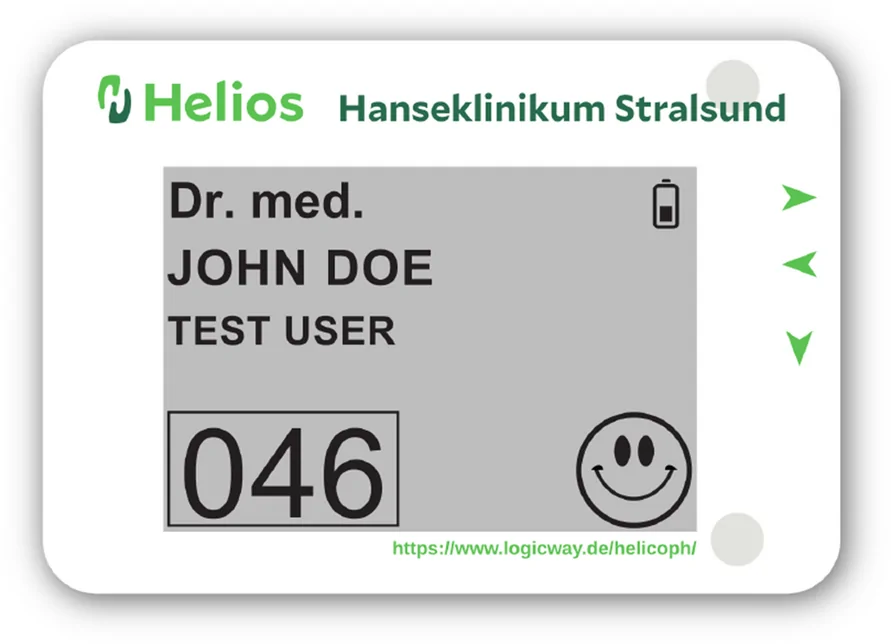
Name badge with exemplary data

Each employee is equipped with their own personalized smart name badge (see Fig. 1), which enables the tracking of individual hand disinfections carried out by the medical staff members [6]. A disinfection is assigned to the name badge that is closest to a disinfectant dispenser at the moment the dispenser is activated. This is achieved by measuring the distance between the dispenser and all name badges in its vicinity using Bluetooth Low Energy Beacon packets.

The integrated e-paper display shows their name and the number of disinfections from the current day at all times. Upon a successfully completed disinfection, a smiley appears in the bottom right-hand corner of the display to symbolize compliance with hygiene regulations [6]. This constant visibility should encourage people to disinfect their hands more often. Furthermore, the absence of the smiley indicating a recent disinfection during patient interactions may negatively affect the patient’s perception of the hygiene standards if they are aware of how the monitoring system operates.

Although intended to demonstrate good hygiene to patients, this mechanism may create undue pressure on the nurses. Failure to comply could trigger stress or a sense of shame, potentially having a negative impact on patient safety. Replacing the counter with a compliance percentage would avoid questions regarding individual diligence but would not solve the core problem. Even with an opt-in system, choosing not to activate the feature could highlight a person as “problematic”. Therefore, hand hygiene monitoring systems should instead focus on supporting staff in maintaining necessary hygiene standards rather than publicly assessing their individual performance. Nevertheless, any decision regarding the system’s design should always be coordinated among hospital management, the works council, and the medical and nursing staff.

### Data management

To ensure privacy, only the timestamps of the performed hand disinfections are stored on the name badge for displaying the cumulative count of disinfections. The radio module on the dispenser does not store any data at all. The data packets that are exchanged between the name badge and the dispenser during the identification process are deleted immediately upon completion.

To enable centralized management, the disinfection data is regularly synchronized with a server via gateways. These gateways are strategically located in areas of the hospital with frequent staff presence to ensure the correct and complete transmission of the data. This exchange occurs automatically whenever a name badge is in proximity to such a gateway, requiring no user interaction.

Through a dashboard, nursing staff members can access their own data and receive direct feedback on their disinfection behavior [6]. While aggregated overviews of data from the entire ward or for a group of people (e.g. all doctors or all nurses) in the hospital are also available, individual data of the colleagues remain strictly inaccessible, even to the management. These statistics, in conjunction with the number and progression of cases of illness, particularly HAIs, allow long-term assessment of the system’s effectiveness at a later date [6].

### Previous observations from the initial pilot phase

The Helicoph system has been in operation in a unit at the Helios Klinikum in Stralsund since December 2018. Following an initial ten-month pilot phase, the clinic published its observations in [6]. Prior to the introduction of the system, the disinfection rate for December 2018 was estimated at 43.95 disinfections per patient-day, which was calculated based on the unit’s total disinfectant consumption [6]. In September of 2019, with the Helicoph system being active for ten months, 62.28 disinfections per patient-day were successfully recorded by the staff members who actively utilized the name badges of the monitoring system [6].

During the same time period, the number of hospital-acquired infections was also tracked. [6] states, that before the installation of the monitoring system, 33 HAIs were reported in December 2018. Ten months later, in September of 2019, only 19 HAIs have been registered in the same hospital unit [6]. In addition to that, [6] argues that the improved hygiene compliance also contributed to a reduction in antibiotic usage and shortened the average length of stay for patients in the intensive care unit.

Direct comparability of these measurements is constrained, however, because the study [6] does not specify the total amount of patient-days and admissions, as well as the adaption rate of the staff. It also does not account for possible seasonal variation or concurrent infection-control interventions in the HAI counting process, which may have an effect on the presented measurements. In addition to that, comparability is further constrained by methodological differences in the disinfection tracking process. The consumption-based estimate covers the total amount of disinfections across the hospital unit, including those from staff, patients and visitors. In contrast to that, the counted disinfections by the system only contain the disinfections performed by the medical staff wearing the name badges. Further empirical research is therefore required to validate these findings.

### Encountered problems

In real-world deployment, the identification process occasionally encounters errors. The signal strength of Bluetooth Low Energy packets is an unstable metric for determining the distance between two devices, as it varies greatly. This instability stems from several factors, such as signal attenuation by the human body or the variation due to premature movement away from the disinfectant dispenser before the identification process is completed [36].

Another limitation is the absence of a reminder function that actively asks the person to perform hand disinfections when an indication is present. The WHO defines five key “moments” for hand hygiene, including immediately before and after patient contact, as well as after leaving the patient environment [4]. Because the smart name badge lacks awareness about the environment, it currently cannot actively notify the person to carry out a hand disinfection when needed. Other scenarios that require hand disinfection include the preparation of syringes or infusions in sterile environments and direct contact with potentially infectious materials [4].

The latter, in particular, is not tied to a fixed location (such as the patient’s bed), but can occur anywhere within the medical facility. The primary technical challenge now lies in accurately detecting whether a nurse has actually performed one of these specific actions. However, this cannot be achieved by simply determining the location, as a nurse might enter a medication room or simply walk past an area without actually engaging in the required activity. To prevent the issuance of false positives – which significantly erodes user acceptance [21] – the system must be able to accurately differentiate between the presence and actual engagement in a hygiene-sensitive task.

### DiSH-O-Klin – extension of the Helicoph system

Based on the results and the problems identified in Encountered problems section, the DiSH-O-Klin research project focuses on the functional expansion and optimization of the Helicoph system [36]. Beyond minor refinements such as revising the name badge attachment options, the project emphasizes significant technical enhancements, which include the implementation of a reminder function. Furthermore, these new developments are designed with careful attention to the ethical considerations and privacy safeguards discussed in Ethical conflict – a first glimpse section.

#### Reminder for a disinfection in certain places

To comply with the WHO guidelines for hand disinfection, the hands must be disinfected when entering and leaving specific clinical areas. But sometimes, this may be forgotten, which has a negative impact on hygiene. To prevent this from happening, a reminder function should be integrated, which actively reminds the medical staff in certain situations to perform a necessary hand disinfection. This would also prevent the monitoring effect from wearing off, which has been proven to cause compliance to decline over time [37].

Implementing this requires the name badge to be aware of its spatial context. One method involves installing motion detectors at key locations in the building [36]. When movement is detected, these sensors broadcast their location information to the name badge via Bluetooth Low Energy beacons, allowing the name badge to estimate its position in the building. Since the badge does not transmit any data back to the system, this process remains entirely anonymous. However, coarse localization is insufficient for events requiring higher precision, such as distinguishing between individual bedsides in a room with multiple patients. In such cases, fine-grained localization with the help of radar sensors would be required.

#### Improving user identification at the dispenser

Erroneous identification of the person that carried out a disinfection not only results in incorrect data records but also significantly erodes user acceptance of the system if such errors occur too frequently [11, 21]. To improve identification accuracy, one approach is to measure the distance between the dispenser and the name badges using acoustic signals [36]. To maintain privacy, the data required for the identification process are generated and handled locally on the involved devices and subsequently discarded. To prevent people in the vicinity of the dispenser from being disturbed, ultrasound frequencies can be used, which are inaudible to humans.

#### Thorough application of the disinfectant

A hand disinfection must always be carried out with great care to ensure that hands are truly hygienically clean. To ensure the disinfectant is optimally and effectively distributed across the entire palm of the hand, the WHO has developed a sequence for disinfecting hands correctly [4]. However, this sequence is not always strictly adhered to, which may allow pathogens to persist.

The research project investigates technical methods to determine whether nursing staff have actually performed the correct hand hygiene movements [36]. While the name badge already provides haptic feedback once the prescribed time for disinfecting the hands has elapsed, it cannot verify whether the required hand movements were correctly executed.

Potential technical implementations to solve this problem include evaluating movement data via wristband-based accelerometers or using optical recognition. However, the latter, in particular, poses a significant risk to privacy due to the use of cameras. Therefore, controlling the disinfection application process should be restricted to training purposes only.

#### Associated ethical risks and proposed safeguards

The expansion options discussed in this section not only list the additional sensors or infrastructure required on the name badge and within the hospital, but also the theoretical data sets generated by these systems. Table 1 combines the ethical risks associated with these technical enhancements with corresponding mitigation strategies and technical safeguards.

Based on current architectural planning, all proposed features can be implemented without the persistent storage of the data points that will be captured in the process. Specifically, motion-based reminder logic, acoustic signal evaluation, and hand-movement analysis can be executed via edge computing directly on the local devices. No storage on a centralized server is needed to provide the mentioned functionalities. For instance, real-time disinfection reminder notifications upon entering a patient room can be generated and processed entirely locally on the deployed hardware in the room and on the name badge.

While logging the metadata regarding the occurrence, location, time, and the compliance with, for example, the disinfection reminder is not strictly necessary for the operation, such data points may be recorded anonymously and stored using robust encryption to support retrospective evaluation on a hospital-wide level. Access to these (aggregated) data points needs to be regulated, documented and limited to the purpose of giving feedback to the medical staff to prevent misuse.

**Table 1:** Overview about potential ethical risks of the technical enhancements and the proposed safeguards

Technical functionality	Associated ethical risk	Proposed safeguards
Motion/Location-Based-Reminders	Infringement on Autonomy	Edge processing
	Perceived surveillance	Anonymized evaluations
	Behavioral manipulation	Documented access
	Unauthorized data exposure	
Improved User Identification with Acoustics	Perceived surveillance	Edge processing
	Loss of privacy	Purpose limitation
Control of the disinfection application process	Infringement on Autonomy	Limited to training purposes
	Surveillance	
	Loss of privacy	

## Conclusion

This paper examines the extent of the ethical conflict between the implementation of a hand hygiene monitoring system and maintaining a good work environment for nursing staff. While German law permits such monitoring depending on the design of the system, it should only serve as assistance rather than a punitive instrument to prevent the deterioration of the good work environment.

Initial observations with Helicoph suggests that digital hand hygiene monitoring can improve hand hygiene compliance and may help reduce hospital-acquired infections (see Previous observations from the initial pilot phase section), though further empirical analysis is needed. At the same time, these findings refer to an earlier version of the system that included visible individual feedback through the name badge. Subsequent design adjustments should therefore respond to some of the ethical concerns identified in this paper, including by shifting the system away from public individualized performance display and toward more supportive forms of assistance, such as reminder functions. Whether this revised design achieves comparable effects remains to be evaluated in a further study.

The development of hand hygiene monitoring systems is still an ongoing and evolving process. While many systems perform well in theory, the complexities of daily clinical workflows present obstacles that these systems must overcome to achieve full acceptance among medical staff [21]. Systems with suboptimal accuracy risk diminishing motivation and eroding trust over time, thereby devaluing the intended benefits of such a system. Future research should therefore also focus on the precise detection of situations requiring hand disinfection and on ethically appropriate ways of reminding nursing staff, so that such systems can contribute both to patient safety and to a good work environment.

## Data Availability

The results of the Helicoph hand hygiene monitoring project were taken from a report which is publicly available: https://management-krankenhaus.de/de/top-themen/innovatives-hygienesystem-reduziert-krankenhausinfektionen.

## Abbreviations

DiSH-O-Klin: **Di**gitale **S**timulation von **H**ygiene- und **O**ptimierungsmaßnahmen im **Klin**ikbetrieb
GDPR: General Data Protection Regulation
HAI: Hospital-Acquired Infection
Helicoph: **HEL**ios **I**nternal **C**ompliance **OP**timization **H**and hygiene
RKI: Robert Koch-Institut
TDoA: Time Difference of Arrival
WHO: World Health Organization

## Footnotes

1 **Di**gitale **S**timulation von **H**ygiene- und **O**ptimierungsmaßnahmen im **Klin**ikbetrieb

2 Funded by the Bundesministerium für Forschung, Technologie und Raumfahrt in Germany

3 **HEL**ios **I**nternal **C**ompliance **OP**timization **H**and hygiene

4 https://www.umg.eu/news-detail/news-detail/detail/news/haendehygiene-digital-verbessern-studie-an-der-universitaetsmedizin-goettingen/

5 https://www.gesetze-im-internet.de/englisch_betrvg/englisch_betrvg.html#p0503
